# Good Clinical Laboratory Practices Improved Proficiency Testing Performance at Clinical Trials Centers in Ghana and Burkina Faso

**DOI:** 10.1371/journal.pone.0039098

**Published:** 2012-06-29

**Authors:** Faisal Ibrahim, David Dosoo, Karl C. Kronmann, Issa Ouedraogo, Thomas Anyorigiya, Haruna Abdul, Sirima Sodiomon, Seth Owusu-Agyei, Kwadwo Koram

**Affiliations:** 1 United States Naval Medical Research Unit -3 Ghana Detachment, Accra, Ghana; 2 Kintampo Health Research Center, Kintampo, Ghana; 3 Center National de Recherche et de Formation sur le Paludisme, Ouagadougou, Burkina Faso; 4 Navrongo Health Research Center, Navrongo, Ghana; 5 Noguchi Memorial Institute for Medical Research, Legon, Ghana; Johns Hopkins University, United States of America

## Abstract

**Background:**

The recent drive towards accreditation of clinical laboratories in Africa by the World Health Organization – Regional Office for Africa (WHO-AFRO) and the U.S Government is a historic step to strengthen health systems, provide better results for patients and an improved quality of results for clinical trials. Enrollment in approved proficiency testing (PT) programs and maintenance of satisfactory performance is vital in the process of accreditation. Passing proficiency testing surveys has posed a great challenge to many laboratories across sub-Saharan Africa. Our study was aimed at identifying the causes of unsatisfactory PT results in clinical research laboratories conducting or planning to conduct malaria vaccine trials sponsored by the National Institutes of Health (NIH).

**Methodology:**

PT reports for 2009 and 2010 from the College of American Pathologists (CAP) for the laboratories were reviewed as part of the process. Errors accounting for unsatisfactory results were classified into clerical, methodological, technical, problem with PT materials, and random errors. A training program on good clinical laboratory practices (GCLP) was developed for each center to address areas for improvement.

**Results:**

The major cause of PT failure in the four centers was methodological. The application of GCLP improved the success rate in the PT surveys from 58% in 2009 to 88% in 2010. It also decreased the error rate on PT by 35%.

**Conclusion:**

A previous report from the CAP- PT participating laboratories indicated that the major causes of error were clerical. These types of errors were predominantly made in laboratories in the US, with much more experience in quality control, and varied significantly from what we found. In our centers in sub-Saharan Africa, methodological errors, and not clerical errors, accounted for the vast majority of errors. A process was started for continuous improvement which has decreased methodological errors by 35%, but more improvement is needed.

## Introduction

There are, currently, a large number of clinical drugs and vaccines trials being conducted in developing countries [1]. Sponsors often face difficulties in maintaining quality system activities in resource limited settings, and in locations where there are no official government or laboratory network standards, and no third party responsible for inspections to maintain compliance [1]. Laboratory auditing by monitors focused on compliance with standard operating procedures (SOPs), will only reflect a “snap shot” appraisal rather than providing continuous training and improvement of day to day activities [2].

The recent drive towards accreditation of laboratories in developing countries by the World Health Organization Regional Office for Africa (WHO-AFRO) [3] is a historic step to strengthen health systems, provide better results for patients, and improve the quality of results for clinical drugs and vaccines trials. Laboratories across Africa are making progress with the WHO-AFRO step-wise approach toward accreditation. Factors that contribute to a successful accreditation project are implementation of Good Clinical Laboratory Practices (GCLP) and the application of a Quality Management System. This includes the enrollment in approved proficiency testing (PT) programs and maintenance of satisfactory performance.

In addition to the provision of equipment from the developed countries, a resolute effort should be made to provide “hands-on” training to maintain a quality system [2]. Over the past few years, the Division of Microbiology and Infectious Disease (DMID) of the National Institutes of Health (NIH) in the United States has been working toward improving the performance of clinical research laboratories of institutions conducting NIH-sponsored clinical trials to ensure that results generated from studies will be reliable and acceptable to regulatory bodies. The ultimate goal of the Quality Assurance/Quality Control (QA/QC) activities described in this paper is to achieve compliance with the College of American Pathologists (CAP) and WHO-AFRO checklists in preparation for accreditation through the implementation of GCLP and the improvement of PT performance.

In PT or External Quality Assessment (EQA), samples are sent out periodically to registered laboratories to be analyzed and/or identified. Results from each laboratory are compared with those of the other participating laboratories in the group [4], [5] for quantitative results like creatinine concentration, or with a pre-determined correct response for qualitative answers such as blood parasite identification. Among other benefits, PT enhances patient care and safety through improved laboratory practice, helps in identifying clinical laboratories that are at risk of performing poorly, and satisfies accreditation and regulatory requirements [5]. Individual laboratories can use PT as an aid to a continual improvement process, by analyzing any substandard results and instituting corrective actions.

The laboratories of the Noguchi Memorial Institute for Medical Research (NMIMR), Navrongo Health Research Center (NHRC) and Kintampo Health Research Center (KHRC) (all in Ghana) and the Center National de Recherche et de Formation sur le Paludisme (CNRFP) in Burkina Faso have been collaborating with the DMID in epidemiological studies and/or vaccine trials. These laboratories have been registered with the CAP for hematology, clinical chemistry and blood parasite surveys as a step towards continual improvement and accreditation. However, the laboratories had difficulties in consistently obtaining satisfactory scores at the beginning. Our aim was to identify the causes of unsatisfactory scores in the CAP PT surveys and to put in place measures to address these causes to improve performance in subsequent surveys.

## Methods

A QA/QC advisor was hired in September 2009 by the U.S Naval Medical Research Unit No. 3 to coordinate the activities of all four trial centers. All PT results obtained in 2009 by the trial centers for General Chemistry, Hematology and Blood Parasites were reviewed, retrospectively. A standardized form was distributed to assist the centers with the investigational process (Table 1) [6]. PT failures were classified into five main categories: clerical, methodological, technical, PT materials stability, and random errors, according to criteria published by the CAP (Table 2) [6]. The contributing causes of unsatisfactory results in 2009 and 2010 are compared with CAP overall PT Surveys of discordant results [6].

**Table 1:** PT investigation aid.

Checklist of items for possible review
1 - Instrument printouts/sheet agrees with submitted information?
2 - Correct unit of measurement and decimal point?
3 - Correct user group/method listed on submitted information/report?
4 - Previous PT results show similar problem/shifts/trends?
5 - QC result for 1 month before and after PT event show evidence of problems/shifts/trends?
6 - QC record show changes of reagents, lot numbers or controls around the time of the survey?
7 - Reagent and controls within expiration date?
8 - Any other failures in this set?
9 - Any training needs identified during discussion?
10 - PT materials were retested and found to be accepted?
11 - Consultation with manufacture indicates matrix effect on the samples?
12 - Tech. re-read SOP (test method + Q.C procedure + reagent Handling) to confirm test method validity?
13 - Follow maintenance table?
14 - Last linearity of device was accepted?
15 - PT materials investigation (handling, storage, analysis sequence, re-constitution and matrix effect)?

**Table 2:** Classification of errors.

Error	Cause(s)
Clerical	1 - Erroneous transcription of results from an instrument print-out or manual log
	2 - Reporting an incorrect unit of Measurement
	3 - Reporting of an incorrect method or instrument
	4 - Misplacement of a decimal point
	5 - The selection of an incorrect reporting code
Methodological	1 - Inappropriate use of QC materials
	2 - Using QC limits that are too wide
	3 - SOP lack guidance on frequency of calibration
	4 - Instrument used without performing test method validation
	5 - Reagent problems
	6 - Poorly written SOPs
	7 - Procedure not in accordance with current standard of practice
	8 - Lot-to-lot variation
	9 - Inadequate maintenance
Technical	1 - Inappropriate sample handling
	2 - Failure to calibrate pipettes
	3 - Inappropriate dilution
	4 - Water quality issues
	5 - Improper reconstitution, preparation or mixing of PT materials
	6 - Microscopic misinterpretation
Stability of PT	1 - Improper storage conditions and/or delay in receiving
Random	1 - Any error that does not fall into any of the above categories

### QA/QC Activities

In order to improve the PT performance for the calendar year 2010, the following GCLP and quality systems measures were taken:

#### Methodological errors

We consulted users of various chemistry equipments in our countries and evaluated their performance in PT schemes, ease of use and maintenance prior to the purchase of new equipment. Uninterruptible Power Supply (UPS) systems were installed for all equipment to ensure stable power during operation. Subsequently, installation qualification (IQ), operator qualification (OQ) and performance qualification (PQ) were performed. The equipment qualification processes ensure that equipment is installed properly, operates as intended by the manufacturer, and continues to be suitable for its intended use. The procedures carried out IQ, OQ, and PQ included inter-equipment comparison, linearity, reproducibility & repeatability (R&R) and stability of control materials. Standard operating procedures for using and maintaining the equipment were prepared using information in the user manuals. Users were given adequate training on operation and user maintenance. A full-time biomedical engineer was employed for servicing the equipment. Levy-Jennings charts were reviewed to determine the proper frequency of calibration intervals. To ensure reagents used for our analysis were properly stored, surprise visits were paid to the suppliers and the importance of storing reagents at the required temperatures was explained to them.

#### Clerical errors

We designed a process to verify clerical entries prior to final approval of PT results. The results were transcribed from instrument print outs to the PT answer sheet by one of the laboratory staff. Then a second laboratory staff will record the results from the answer sheet to the CAP website. Subsequently, the unit head or designee reviewed the entered results against the instrument print-outs.

Laboratory staff were advised to (1) select appropriate reagents, instrument and method codes; and to modify them if change of reagent, instrument or method occurred before the next PT shipment, (2) select the correct unit of measurement for each result entered, (3) select the correct code if the laboratory is not able to perform testing due to instrument malfunction or reagents shortage, and (4) ask for extension in rare occasions when the laboratory is not able to meet the deadline.

#### Technical errors

A one-week refresher training on preparation of quality blood smears and identification of blood parasites was organized for all of our centers at the Malaria Diagnostic Center, KHRC in Ghana. The malaria diagnostic center of KHRC was established as a center of excellence in 2008 as collaboration between KHRC, the Walter Reed project (Kenya) and the Malaria Clinical Trials Alliance (MCTA). KHRC conducted this training for the other centers before the first survey for blood parasites was received in 2010. During the training, microscopists shared their SOPs and discussed many issues related to malaria microscopy, such as QA/QC in the collection and preparation of blood smears, parasite identification and quantification. Pre- and post- test performances for each microscopist were evaluated.

The need to follow specimen handling instructions that come with the PT kit, use of calibrated pipettes and high purity water in preparation of chemistry reagents, calibrators and control materials was discussed with laboratory staff. For quantitative tests, we monitored the standard deviation index (SDI) from the PT summary and Participants’ Summary in relation to our test results. We aimed at having the SDI as close as possible to zero and our QC results as close as possible to the mean. If both SDI and QC results indicate high or low results then calibration or adjusting the calibration factor can resolve the issue. A high QC/SDI result means a QC value above +1SD and all five PT samples yielding positive SDI. A negative QC/SDI means a QC value below -1SD and all five PT samples yielding a negative SDI. However, if there was a positive SDI with a low QC results, the biomedical engineer and supplier were consulted, and they performed careful investigation. With the blood parasites PT, we redistributed misidentified slides or photographs to the microsocopists after grading of the challenge to allow them to have a second look. The participants’ summary discussion was used as an in-service training aid.

Extensive reviews of the user manuals for our equipment were performed. In addition, SOPs for the maintenance and daily operation of the equipment were written and made available to all personnel.

#### PT stability

Our laboratories communicated with the airport officials the importance of keeping our samples at acceptable temperature during the paperwork clearance process. Moreover, if a shipment did not arrive within five days of the scheduled shipping date, a follow up process would be initiated. Usually, the PT vendor provided our laboratories with a tracking number to locate the shipment during transit. Once the shipment arrived, it would be analyzed as soon as possible.

#### Random errors

When all the aforementioned causes of errors were excluded, we would classify the cause of failure as random error, especially when repeat testing indicated an acceptable performance. Our laboratories would not perform any corrective actions when random error was identified. Adjusting the testing system due to random errors can lead to future failures.

## Results

**Figure 1:**
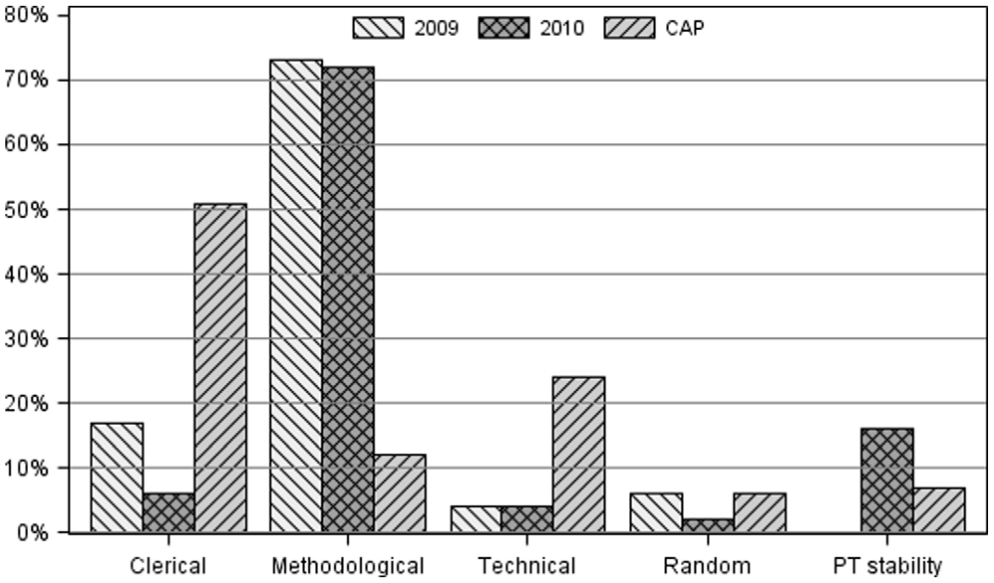
Comparison of proportion of errors due to each type of error for unsatisfactory results at study centers in 2009, 2010, and US averages according to CAP data.

Methodological errors accounted for the majority of unsatisfactory results for both years in our laboratories (Figure 1). There was a 33% increase in PT conducted by the four centers (24 in 2009, 32 in 2010), and therefore more opportunities for error. However, despite the increase in testing, there was a 12% decrease in the absolute number of errors, from 180 in 2009 to 159 in 2010, and the error rate per survey decreased by 35%. The greatest reduction in the number errors was for clerical errors, from 31 errors in 2009 to 10 errors in 2010, while methodological errors decreased from 131 to 115 (Table 3). Most of the methodological errors were from quantitative test results. The majority of quantitative errors occurs due to inappropriate or lack of calibration. However, while there was no error due to PT stability in 2009, this increased to 16% (n = 25) in 2010 (Table 3). Overall, the error rate per survey dropped from 7.5 error/survey (180/24) in 2009 to 4.9 errors/survey (159/32) in 2010, a 35% reduction.

**Table 3:** PT error rates in 2009–2010 for all centers.

Year	Methodological	Technical	Clerical	PT Stability	Random
2009	73% (131)	4% (8)	17% (31)	0	6% (10)
2010	72% (115)	4% (6)	6% (10)	16% (25)	2% (3)
2009/2010 average	73% (246)	4% (14)	12% (41)	7% (25)	4% (13)
CAP (2007)	12%	24%	51%	7%	6%

**Figure 2:**
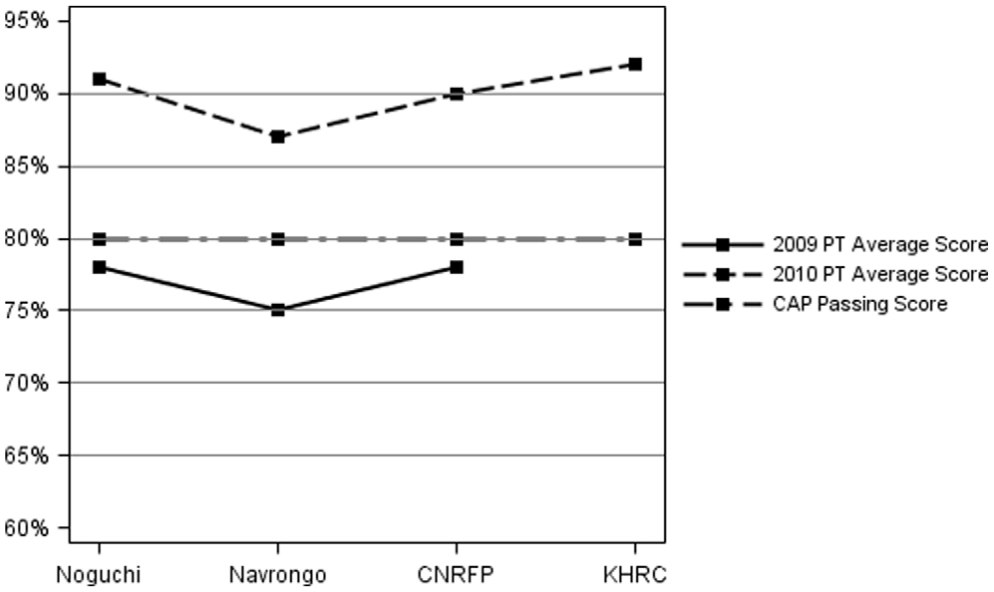
Average PT performance by site.

PT survey success rate in 2009 was 58% (14 of 24 PT shipments) while in 2010 the rate increased to 88% (28 of 32 PT shipments) (Table 4). Average performance scores for all centers in 2009 and 2010 (Figure 2) were 77% and 90%, respectively (CAP cutoff for satisfactory results is 80%). Site-specific average performances are shown in Figure 2.

**Table 4:** PT Performance in 2009–2010 for all centers.

Year	PT success rate	Average PT score
2009	58% (14 out of 24 surveys)	77%
2010	88% (28 out of 32 surveys)	90%

**Figure 3:**
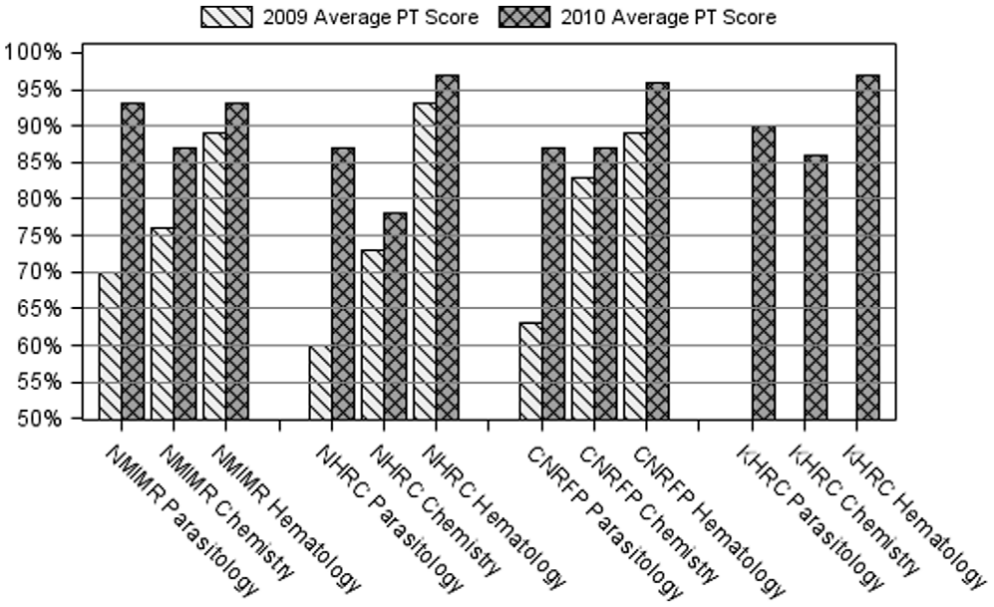
Performance by Center for 2009 and 2010 for blood parasites, chemistry and hematology.

Figure 3 shows how each site performed in the different areas of testing in 2009 and 2010. Apart from the chemistry PT for NHRC, all other centers recorded an average score of more than 80% in the 2010 PTs, an improvement over the previous year’s performance. There was no data for KHRC in 2009 because testing began 2010.

**Figure 4:**
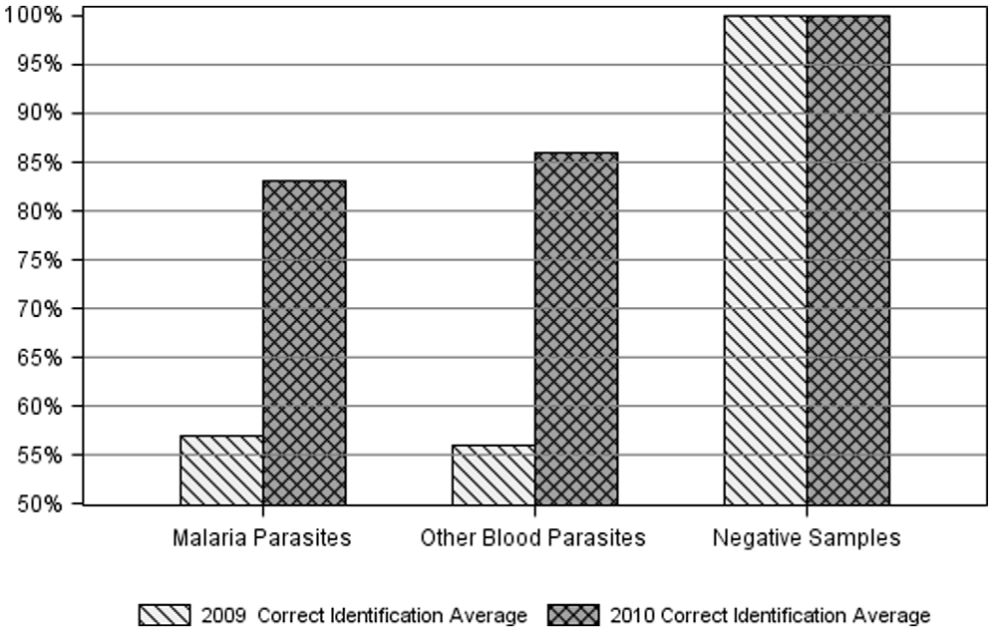
Average correct identification of the blood parasites surveys for all sites.

Figure 4 shows the average correct identification of malaria parasites, other blood parasites, and negative slides for all sites. Both malaria parasites and the other blood parasites improved significantly between 2009 and 2010 (57% to 83% and 56% to 86% respectively). All negative slides were identified correctly in both years.

Our surprise visits to reagent suppliers revealed poor storage conditions. Of the four suppliers visited, only one had temperature records. The recorded temperatures for the refrigerators were higher than the recommended 2–8°C due to overloading of the refrigerators. Room temperatures were higher than the recommended 20–25°C, as there was no air-conditioning in the reagent storage rooms.

## Discussion

Investigating causes of unsatisfactory performance and the immediate application of interventions are crucial steps in preventing future occurrences. Methodological errors were the major cause of unsatisfactory results in our laboratories. A previous report from the CAP PT participating laboratories indicate the possible causes of errors were 51% clerical, 24% technical, 12% methodological, 7% problem with PT materials, and 6% random errors [6]. These types of errors were predominantly made in laboratories in the US, with much more experience in quality measures, and varied significantly from what we found. In our centers in sub-Saharan Africa, methodological errors, and not clerical errors, accounted for the vast majority of errors. We suspect this reflects the lack of experience of many laboratory staff in developing countries with quality control methods used in developed countries. Through ongoing training and evaluation of performance on PT testing, we were able to decrease the number of methodological errors per PT from 5.5 errors/PT in 2009 to 3.6 errors/PT in 2010, a 35% reduction in methodological errors.

Specific items that were noted to contribute to failures include equipment selection, test method validation, equipment maintenance, reagent quality and storage, quality control procedures, continuing education, availability of expertise, and PT stability. Each of these items is discussed below:

### Equipment Selection

Equipment related problems contributed greatly to the methodological errors. Proper selection and acquisition of the right equipment is the first step for a successful journey to quality [7]. We often face the dilemma of purchasing open-system equipment which requires highly skilled staff versus closed system equipment which often places the laboratory at the mercy of an unreliable vender to provide reagents on time. According to Petti *et al* (2006), only 26% of laboratory staff in Ghana are professionally qualified [8]. Against this background, we performed extensive user manual review, technical, and refresher trainings.

### Test Method Validation

Once equipment is purchased, laboratories should ensure test method validation (TMV) is performed. TMV includes accuracy, precision, analytical sensitivity, analytical specificity, reportable range, and reference intervals [9]. Unfortunately, in our setting, the role of suppliers has been limited to the installation and basic user training. This is similar to what was reported by Crucitti and colleagues (2010) [2]. It is, however, the responsibility of each laboratory to ensure the completion and acceptance of TMV prior to the initiation of patient testing [10].

### Equipment Maintenance

Once equipment is installed, laboratories should ensure proper scheduling, performance and documentation of daily, weekly, monthly, and other recommended maintenance [9]. Integrating the user’s manual into the SOP has simplified the steps needed to accomplish assigned tasks consistently. The presence of the full-time biomedical engineer ensures proper performance and monitoring of required maintenance which lessen equipment downtime. Furthermore, due to fluctuations in the supply of electricity an un-interruptible power supply system was always provided.

### Reagents

Poor storage and transport of reagents, controls and calibration materials by suppliers often leads to poor reagent performance that negatively impact the quality of testing. To eliminate these effects, our trial centers now procure reagents from reputable suppliers who showed proper storage condition and documentation. It is important that laboratories work with suppliers in improving service performance [11].

### Quality Control

QC must be tested and acceptable results obtained prior to release of patients results [9], [12]. All centers were trained in QC monitoring, real-time plotting of Levy Jennings charts and the implementation of statistical QC rules [13]. Laboratory supervisors were trained on how to calculate and implement in-house control values as ranges provided by manufacturers tend to be too wide [9]. The adaptation of in-house tighter control ranges assisted our centers in the early detection of accuracy and precision related problems before it affects participants’ results and/or PT samples.

### Continuing Education

Some of the errors encountered in the blood parasites surveys came from non-malaria organisms such as *Babesia*, *Leishmania*, and microfilaria (pre-larval stage) which are not commonly seen in our centers. The malaria microscopy refresher training provided laboratory microscopists from all centers an opportunity for interaction and experience sharing. Networking and exchange of information among scientists in resource limited settings is vital [14]. Scheduling regular refresher training among scientists will assist our centers to overcome “intellectual isolation” [15]. Improving accuracy of malaria microscopy can avert the recent trend of Ghanaian physicians who use empirical methods to diagnose malaria [16].

### QA/QC Expertise

The presence of a QA/QC advisor provided the centers with the hands-on technical support needed during clinical trials. Most of the supervisors in resource limited settings lack the technical expertise necessary to ensure accuracy of test results [8]. It is obvious that establishing a quality assurance program will increase the trial budget [2]. However, these activities will ensure the safety of participants, accuracy of testing and subsequently improve healthcare in resource limited settings [2]. According to Simon [17], programs measure their success by factors such as: the number of publications, work cited, number of funded studies, and number of scientist trained. Sustaining successful performance in proficiency testing should be used as a direct indicator of the quality of laboratory performance.

### PT Stability

We did not experience unsatisfactory performance as a result of unsuitable PT samples in 2009. This was, however, a significant contributing factor to the errors encountered in 2010. The PT stability error rate was about two times the value of the CAP data. Issues with PT survey materials stability are common in international laboratories. Many times the PT materials travel through several airports and warehouses before they arrive at their final destination. The temperature during the travel is not monitored and/or controlled. Some of the PT materials will require customs clearance before or after its arrival. Ensuring proper storage conditions during the paperwork processing is crucial to the stability of the PT materials. Some postal carriers, who lack refrigeration facilities at the airport, can accept refrigerators donated by laboratories to be used for storage of their shipment. However, this step will require a complete understanding from the postal carriers of the importance of compliance with temperature requirements. Laboratories should monitor the shipping calendar closely. Any delays of samples testing can negatively impact the quality of results. International laboratories do not have to order the PT product with less than ten days of stability [18]. The PT ordering booklet contains the list of tests with less than ten days shelf life. An alternative assessment should be performed instead [19].

### Conclusion

The intervention impact has been positive. All our centers had a higher average performance score in 2010 compared to that of 2009. KHRC did not have scores for 2009 because participation in the PT surveys started in 2010.

Improving the performance of laboratories in PT is a continuous process. Through a continuous program of education and training described above, we were able to achieve a 35% reduction in errors over the course of a year. However, despite a 35% reduction in methodological errors, this type of error remains the most common cause of errors at the four clinical trial centers in sub-Saharan Africa, accounting for 73% of errors. Comparing this rate to the US rate of methodological errors of 12%, emphasizes the need for more training of laboratory staff in this region in quality control methods that are well established in developed countries. Our centers will continue to use PT to improve Quality Assurance practices in our laboratories. We look forward to a continuous improvement process and sharing our experiences with other laboratories in the region.
